# Health related quality of life in patients with idiopathic pulmonary fibrosis in clinical practice: insights-IPF registry

**DOI:** 10.1186/s12931-017-0621-y

**Published:** 2017-07-14

**Authors:** Michael Kreuter, Jeff Swigris, David Pittrow, Silke Geier, Jens Klotsche, Antje Prasse, Hubert Wirtz, Dirk Koschel, Stefan Andreas, Martin Claussen, Christian Grohé, Henrike Wilkens, Lars Hagmeyer, Dirk Skowasch, Joachim F Meyer, Joachim Kirschner, Sven Gläser, Felix J. F. Herth, Tobias Welte, Claus Neurohr, Martin Schwaiblmair, Matthias Held, Thomas Bahmer, Marion Frankenberger, Jürgen Behr

**Affiliations:** 1 Center for interstitial and rare lung diseases, pneumology and respiratory critical care medicine, Thoraxklinik, University of Heidelberg, Röntgenstr. 1, D-69126 Heidelberg, Germany; 2 Interstitial Lung Disease Program, National Jewish Health, Denver, CO USA; 3 Institut für Klinische Pharmakologie, Medizinische Fakultät, Technische Universität Dresden, Dresden, Germany; 4 Department Market Access, Boehringer Ingelheim, Ingelheim am Rhein, Germany; 5 Epidemiologie, Deutsches Rheuma-Forschungsinstitut, Berlin, Germany; 6 Klinik für Pneumologie, Medizinische Hochschule Hannover, Hannover, Germany; 7 Fraunhofer Institute ITEM, Hannover, Germany; 8 Abteilung für Pneumologie, Department Innere Medizin, Neurologie und Dermatologie, Universitätsklinikum Leipzig AöR, Leipzig, Germany; 9 Zentrum für Pneumologie, Fachkrankenhaus Coswig, Coswig, Germany; 10 Lungenfachklinik Immenhausen and Universitätsmedizin Göttingen, Kardiologie und Pneumologie, Göttingen, Germany; 11 LungenClinic Grosshansdorf, Grosshansdorf, Germany; 12 Klinik für Pneumologie – ELK, Berlin Buch, Berlin, Germany; 13 Klinik für Innere Medizin V, Pneumologie, Universitätsklinikum Universitätskliniken des Saarlandes, Homburg, Germany; 14 Krankenhaus Bethanien, Solingen, Germany; 15 Medizinische Klinik und Poliklinik II, Universitätsklinikum Bonn, Bonn, Germany; 16 Lungenzentrum München, LZM Bogenhausen-Harlaching, Städtisches Klinikum München GmbH, München, Germany; 17 Center for Internal Medical Studies CIMS, Bamberg, Germany; 18 Universitätsmedizin Greifswald, Klinik und Poliklinik für Innere Medizin B, Forschungsbereich Pneumologie und Pneumologische Epidemiologie, Greifswald, Germany; 19 Vivantes Klinikum Spandau, Klinik für Innere Medizin, Berlin, Germany; 20 Comprehensive Pneumology Center, Lungenforschungsambulanz, Klinikum der Universität München, München, Germany; 21 I. Medizinische Klinik, Klinikum Augsburg, Augsburg, Germany; 22 Klinikum Würzburg Mitte, Standort Missioklinik, Abteilung Innere Medizin, Pneumologie, Würzburg, Germany; 23 Asklepios Fachkliniken München-Gauting, München, Germany; 24 German center for Lung Research, Aulweg 130, 35392 Gießen, Germany

**Keywords:** Patient related outcomes, Psychometrics, Idiopathic pulmonary fibrosis, Cohort study

## Abstract

**Background:**

The INSIGHTS-IPF registry provides one of the largest data sets of clinical data and self-reported patient related outcomes including health related quality of life (QoL) on patients with idiopathic pulmonary fibrosis (IPF). We aimed to describe associations of various QoL instruments between each other and with patient characteristics at baseline.

**Methods:**

Six hundred twenty-three IPF patients with available QoL data (St George’s Respiratory Questionnaire SGRQ, UCSD Shortness-of-Breath Questionnaire SoB, EuroQol visual analogue scale and index EQ-5D, Well-being Index WHO-5) were analysed. Mean age was 69.6 ± 8.7 years, 77% were males, mean disease duration 2.0 ± 3.3 years, FVC pred was 67.5 ± 17.8%, DL_CO_ pred 35.6 ± 17%.

**Results:**

Mean points were SGRQ total 48.3, UCSD SoB 47.8, EQ-5D VAS 66.8, and WHO-5 13.9. These instruments had a high or very high correlation (exception WHO-5 to EQ-5D VAS with moderate correlation). On bivariate analysis, QoL by SGRQ total was statistically significantly associated with clinical symptoms (NYHA; *p* < 0.001), number of comorbidities (*p* < 0.05), hospitalisation rate (*p* < 0.01) and disease severity (as measured by GAP score, CPI, FVC and 6-min walk test; *p* < 0.05 each). Multivariate analyses showed a significant association between QoL (by SGRQ total) and IPF duration, FVC, age, NYHA class and indication for long-term oxygen treatment.

**Conclusions:**

Overall, IPF patients under real-life conditions have lower QoL compared to those in clinical studies. There is a meaningful relationship between QoL and various patient characteristics.

**Trial registration:**

The INSIGHTS-IPF registry is registered at Clinicaltrials.gov (NCT01695408).

**Electronic supplementary material:**

The online version of this article (doi:10.1186/s12931-017-0621-y) contains supplementary material, which is available to authorized users.

## Background

Idiopathic pulmonary fibrosis (IPF) is a chronic, fibrosing interstitial lung disease associated with a high symptom burden, significant comorbidities and early death [1–3]. Median survival is 3–5-years, shorter than for many malignancies [4]. The antifibrotic drugs, pirfenidone and nintedanib, slow lung function decline but have not been convincingly shown to improve survival or quality of life (QoL) [5, 6]. Beside prolonging survival, major aims for IPF therapy include improving symptoms and QoL domains like physical functioning, social participation and emotional well-being [7].

A number of patient-reported outcome (PRO) measures have been used in IPF research [8]. However, the majority of PRO data were generated in single-center cohorts or controlled clinical trials, and there are very limited QoL response data from IPF patients collected under real-world conditions. Such data could be used to improve understanding of disease burden at the individual and group levels, to better discern response to therapeutic interventions and to plan for trials of novel therapies.

In the present study, we aimed to summarize QoL data collected in a nationwide, “real-world”, observational registry of patients with IPF and to examine associations between QoL and several other clinical variables.

## Methods

INSIGHTS-IPF (“Investigating significant health trends in idiopathic pulmonary fibrosis”) is an investigator-initiated, multicenter (19 centers from all parts of Germany), observational registry study of data collected, within the confines of routine clinical care, from patients with IPF since November 2012. The study materials were approved by the Ethics Committee of the Medical Faculty, Technical University of Dresden, and by further local ethic committees as per local requirements. INSIGHTS-IPF is registered at Clinicaltrials.gov (NCT01695408). The protocol [9, 10] and a detailed description of the baseline characteristics of the cohort [1] have been previously published. In brief, patients are eligible for enrolment if they are at least 18 years old, have IPF (definite, probable or possible, applying the 2011 IPF guideline [11]) based on physician diagnosis, and have provided written informed consent. There are no explicit exclusion criteria. Clinical data are collected at enrolment and thereafter at 6-month intervals. At follow-up visits, events such as hospitalization and acute exacerbation (as judged by the treating physician) are recorded. Data are reported via a secure internet based data collection form.

### Patient-reported outcome measures

Enrollees complete PROs at enrolment and yearly thereafter. PROs include the University of California San Diego Shortness of Breath Questionnaire (UCSD SOB), the St. George’s Respiratory Questionnaire (SGRQ), World Health Organization-5 Well-Being Index (WHO-5) and the EuroQol five-dimensional questionnaire (EQ-5D).

#### UCSD SOB

This questionnaire includes 24 items, each with a response scale 0 (Not at all) to 5 (Maximally or Unable to do because of breathlessness). The total score ranges from 0 to 120, with a higher score indicating more severe dyspnea [12, 13].

#### SGRQ

The SGRQ was originally developed for patients with chronic obstructive pulmonary disease or asthma [14], however, as a respiratory disease-specific instrument, it has frequently been used in IPF [15]. There are 50 items divided into three components (symptoms, activity, and impacts). Scores for each component and a total score range from 0 (highest QoL) to 100 (poorest QoL).

#### WHO-5

The 5 items of the questionnaire tap mood, vitality, and general health. Each item is scored 0 to 5. The total ranges from 0 to 25, with higher scores connoting better well-being.

#### EQ-5D

The EQ-5D taps 5 domains (mobility, self-care, usual activities, pain or discomfort, and anxiety or depression) and is commonly used in cost-utility evaluation. Based on domain scores, a sum utility score is calculated ranging from negative values (−0.59 worse than death) to 1 (perfect state). Respondents also rate their current health on a 20-cm vertical visual analogue scale (VAS) scored from 0 to 100 [16].

### Data collection and statistical analysis

Data were collected using an internet-based case report form (eCRF) with automated plausibility checks. On-site monitoring, with source data verification, was performed in the majority of centers (currently 70%).

Summary statistics were generated for baseline data. Pearson product-moment correlation coefficients and univariate linear regression were used to examine associations between variables. Backward selection was used to generate multivariable models using the following candidate variable: disease duration, long-term oxygen therapy, physician’s judgment on IPF behavior (stable, slowly or rapidly progressing), NYHA stage, duration since first symptoms in years, GAP index [17], number and type of comorbidities (left heart insufficiency, coronary heart disease (CHD), carotid stenosis, stroke, peripheral arterial disease, atrial fibrillation, deep venous thrombosis (DVT), pulmonary arterial embolism, pulmonary hypertension, arterial hypertension, reflux, diabetes mellitus, emphysema, lung cancer, obstructive sleep apnea, depression/depressive disorder, anxiety), 6-min walk distance, gender, hospitalization in last 12 months, pulmonary rehabilitation, and CPI. Standard errors and confidence intervals were estimated by the Huber White sandwich estimator to account for the clustering of patients within the study centers. Data were analyzed with STATA 12.1 (StataCorp LP. Stata Statistical Software: Release 12. College Station, TX, USA).

## Results

### Baseline characteristics

Data for QoL were available for 623 of a total of 737 patients (84.5%). Baseline characteristics are presented in Table 1. Patients mean age was 69.6 ± 8.7 years, 77.2% were male; all but one were Caucasian (99.7%). Their mean FVC was 67.5 ± 17.8% predicted and DLCO 35.6 ± 17% predicted. A comparison of baseline characteristics of the 623 patients with and 114 patients without available QoL data can be found in Additional file 1: Table S1.

**Table 1:** Baseline characteristics

Characteristic	Value
Male sex	481 (77.2%)
Age, years	69.6 ± 8.7
Body mass index, kg/m^2^	27.5 ± 4.1
Underweight	4 (0.6%)
Normal weight	167 (26.8%)
Overweight	305 (49.0%)
Obesity	147 (23.6%)
Age at first symptom onset, years	65.8 ± 10.1
Age at IPF diagnosis, years	67.6 ± 9.6
Duration since first symptoms, years	3.6 ± 4.0
Disease duration, years	2.0 ± 3.3
Disease duration of less than 6 months	242 (38.8%)
Smoking status	
Never	237 (38.0%)
Former	376 (60.4%)
Current	10 (1.6%)
Gastro-oesophageal reflux	192 (30.8%)
Emphysema	55 (8.8%)
Genetic predisposition	31 (5.0%)
Six-minute walk distance, meters	272.4 ± 196.1
% FVC	67.5 (±17.8)
% FEV_1_	75.3 (±19.4)
% DL_CO_	35.6 (±17.0)
Long term oxygen use	201 (32.3%)
GAP index	
Stage I	87 (20.2%)
Stage II	238 (55.2%)
Stage III	106 (24.6%)

Based on sample of patients with HrQoL data (*n* = 623). Values are n (%) or mean ± standard deviation

*GAP* Gender, Age, Physiology index

Patients were treated with antifibrotic therapies (49.5%), oral glucocorticoids (23.7%); N-acetylcysteine (33.7%), and long-term O2 therapy (32.3%). Most (90.0%) had definite IPF, 5% probable IPF, and 5% possible IPF. At enrolment, treating physicians rated IPF as stable in 36.3%, slowly progressing in 30.9% and rapidly progressing in 11.2%.

### PRO scores and their inter-correlations at enrolment

Baseline values for PROs and their inter-correlations are shown in Table 2. According to the SGRQ, the greatest impairment was in the activity component. Based on the WHO-5 index, 46.4% of the patients showed depressive symptoms.

**Table 2:**
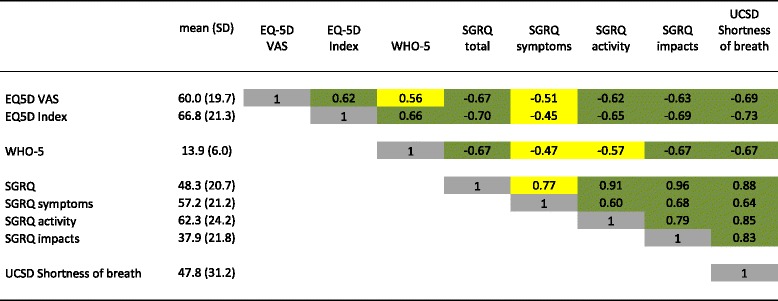
Correlations between different measures of QoL at baseline

Green fields highlight very strong (*r* ≥ 0.80) or strong (*r* ≥ 0.60–0.79) correlations, yellow fields moderate (*r* = 0.30–0.59) correlation

### Associations between PRO scores and clinical variables at enrolment

**Fig. 1:**
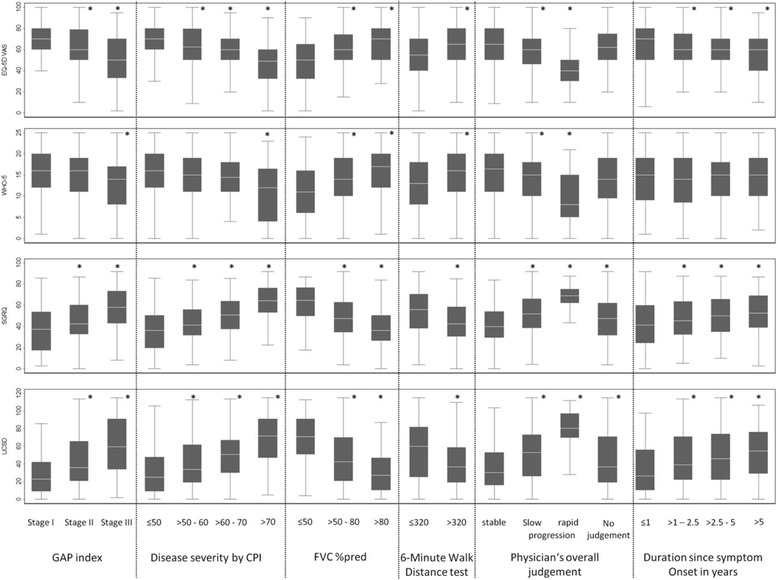
QoL Scores by Disease severity (* *p* < 0.05 in reference to the first category)

For the SGRQ, associations with various demographic and clinical characteristics of patients at baseline are shown in Fig. 1. Statistically significantly higher total SGRQ score (indicating reduced QoL) were associated with lower age (51.8 for patients ≤60 years versus 46.9 for patients >65 years), female gender (46.9 for male versus 53 for female), higher NYHA classes (compared to NYHA class I), longer duration of symptoms, higher CPI, lower %FVC, and higher GAP stage (Fig. 1). Correlations between QoL and %DLCO (EQ-5D: 0.28, *p* < 0.001; SGRQ: -0.26, *p* < 0.001; UCSD: -0.22, *p* < 0.001) or %FVC (EQ-5D: 0.33, *p* < 0.001; SGRQ: -0.40, *p* < 0.001; UCSD: -0.43, *p* < 0.001) were moderately strong. Patients without comorbidity had a mean SGRQ total score of 44; those with 2 comorbidities 47; and those with ≥4 comorbidities 59 (ANOVA, *p* < 0.001 for difference between groups) (Table 3). QoL was also significantly associated with some types of pharmacological and non-pharmacological therapies of patients with IPF (Table 4).

**Table 3:** Association of QoL and comorbidities

		EQ-5D VAS		WHO-5		SGRQ total		UCSD	
	N (%)	mean (SD)	Delta 95% CI *p* value	mean (SD)	Delta 95% CI *p* value	mean (SD)	Delta 95% CI *p* value	mean (SD)	Delta 95% CI *p* value
No comorbidity	151 (20.5%)	65.6 (20.0)	(ref)	15.1 (5.6)	(ref)	44.1 (22.0)	(ref)	55.5 (21.7)	(ref)
One comorbid disease	196 (26.6%)	61.5 (20.2)	−4.05 (−8.65; 0.56) 0.085	14.5 (6.3)	−0.60 (−1.98; 0.78) 0.393	46.1 (21.1)	2.03 (−3.05; 7.11) 0.433	56.0 (21.6)	3.79 (−4.39; 11.96) 0.363
Two comorbid diseases	184 (25.0%)	60.0 (19.1)	−5.54 (−10.09; −0.99) 0.017	14.4 (5.5)	−0.70 (−2.02; 0.63) 0.305	46.8 (19.4)	2.78 (−2.18; 7.74) 0.271	55.8 (20.7)	9.08 (0.62; 17.54) 0.036
Three comorbid diseases	116 (15.7%)	56.0 (17.9)	−9.53 (−14.51; −4.55) <0.001	12.5 (6.1)	−2.61 (−4.23; −0.99) 0.002	52.9 (19.5)	8.80 (3.17; 14.44) 0.002	60.9 (21.7)	19.73 (10.59; 28.87) <0.001
≥Four comorbid diseases	90 (12.2%)	50.9 (18.3)	−14.66 (−20.18; −9.14) <0.001	10.6 (6.0)	−4.54 (−6.26; −2.82) <0.001	59.1 (16.7)	15.03 (9.40; 20.65) <0.001	61.4 (19.5)	31.36 (20.16; 42.57) <0.001
List of significant comorbidities		Left heart insufficiency, Coronary heart disease, Carotid stenosis/ Stroke, Atrial fibrillation, Pulmonary arterial embolism, Pulmonary hypertension, Arterial hypertension, Diabetes mellitus, Emphysema, Lung cancer, Depression, Anxiety		Left heart insufficiency, Coronary heart disease, Carotid stenosis/ Stroke, Atrial fibrillation, Pulmonary arterial embolism, Pulmonary hypertension, Arterial hypertension, Diabetes mellitus, Depression, Anxiety		Left heart insufficiency, Coronary heart disease, Carotid stenosis/ Stroke, Pulmonary hypertension, Lung cancer, Depression, Anxiety		Left heart insufficiency, Coronary heart disease, Carotid stenosis/ Stroke, Pulmonary hypertension, Lung cancer, Depression, Anxiety	

*95% CI* 95% confidence interval, *delta* mean difference between the groups, *ref.* reference group, *SD* standard deviation

**Table 4:** Association of QoL and therapy for IPF

	N (%)	mean (SD)	Delta 95% CI *p* value	mean (SD)	Delta 95% CI *p* value	mean (SD)	Delta 95% CI *p* value	mean (SD)	Delta 95% CI *p* value
Antiinflammatory therapy^a^									
No	426 (57.8%)	63.6 (19.0)	(ref)	14.9 (5.9)	(ref)	43.9 (19.9)	(ref)	40.0 (27.9)	(ref)
Yes	311 (42.2%)	55.4 (19.7)	−8.25 (−11.35; −5.15) <0.001	12.6 (6.0)	−2.22 (−3.18; −1.25) <0.001	53.7 (20.4)	9.84 (6.54; 13.13) <0.001	57.6 (32.3)	17.55 (11.47; 23.62) <0.001
Antifibrotic therapy									
No	372 (50.5%)	59.7 (20.9)	(ref)	14.0 (5.9)	(ref)	46.8 (21.7)	(ref)	46.2 (32.0)	(ref)
Yes	365 (49.5%)	60.3 (18.4)	0.63 (−2.48; 3.74) 0.691	13.8 (6.2)	−0.19 (−1.17; 0.78) 0.698	49.9 (19.4)	3.05 (−0.29; 6.38) 0.074	49.4 (30.3)	3.13 (−3.04; 9.31) 0.319
Long-term oxygen therapy									
No	503 (68.3%)	65.8 (17.9)	(ref)	15.2 (5.7)	(ref)	42.0 (19.2)	(ref)	36.4 (27.3)	(ref)
Yes	234 (31.8%)	47.8 (17.9)	−17.93 (−20.96; −14.90) <0.001	11.1 (5.7)	−4.15 (−5.14; −3.17) <0.001	61.2 (17.3)	19.18 (16.08; 22.28) <0.001	71.2 (24.9)	34.83 (29.40; 40.25) <0.001
Other non-pharmacological therapy^b^									
No	721 (97.8%)	60.3 (19.7)	(ref)	14.0 (6.0)	(ref)	48.1 (20.6)	(ref)	47.5 (31.1)	(ref)
Yes	16 (2.2%)	49.4 (19.1)	−10.86 (−20.35; −1.37) 0.025	10.3 (6.4)	−3.71 (−6.87; −0.54) 0.022	56.3 (23.0)	8.24 (−3.55; 20.04) 0.17	66.4 (30.5)	18.98 (−2.30; 40.25) 0.08
Pulmonary rehabilitation									
Unknown	219 (30.3%)	57.7 (20.5)	−4.04 (−7.57; −0.50) 0.025	13.5 (6.3)	−0.69 (−1.80; 0.42) 0.222	50.8 (21.2)	4.56 (0.78; 8.34) 0.018	52.8 (34.0)	8.32 (1.01; 15.63) 0.026
No	464 (64.3%)	61.8 (19.2)	(ref)	14.2 (6.0)	(ref)	46.3 (20.4)	(ref)	44.5 (29.7)	(ref)
Yes	39 (5.4%)	51.5 (19.4)	−10.24 (−17.14; −3.33) 0.004	11.9 (5.3)	−2.25 (−4.20; −0.31) 0.023	60.7 (17.1)	14.42 (8.20; 20.64) <0.001	67.2 (27.6)	22.63 (10.20; 35.06) <0.001
Reflux therapy									
No	510 (69.2%)	61.8 (19.7)	(ref)	14.3 (6.0)	(ref)	46.4 (20.3)	(ref)	44.5 (30.2)	(ref)
Yes	227 (30.8%)	56.1 (19.3)	−5.67 (−8.99; −2.34) 0.001	12.8 (5.9)	−1.53 (−2.57; −0.48) 0.004	52.7 (20.8)	6.31 (2.67; 9.94) 0.001	54.7 (32.1)	10.13 (3.45; 16.80) 0.003

^a^Daily oral glucocorticoids; *95% CI* 95% confidence interval, *delta* mean difference between the groups, *ref.* reference group, *SD* standard deviation

^b^Other non-pharmacological therapy includes: flutter, physiotherapy, yoga, inhalation furosenid, breathing therapy, spinal exercise, tai chi, cardiac pacemaker

In multivariate models (Table 5), LTOT, GAP index (stage III), physician’s judgement (rapid progression), and NYHA class were independent predictors of EQ-5D VAS. The same variables (except for the GAP index) were associated with SGRQ total score.

**Table 5:** Predictors of QoL in stepwise multivariable linear regression analyses

	EQ-5D VAS	WHO-5	SGRQ	UCSD
	Beta 95% CI *p* value	Beta 95% CI *p* value	Beta 95% CI *p* value	Beta 95% CI *p* value
Age			0.28 0.05; 0.52 0.018	
Disease duration in months			0.07 0.02; 0.12 0.010	
GAP index				
Stage I	(ref)			
Stage II	−5.02 −10.78; 0.74 0.087			
Stage III	−12.24 −19.71; −4.78 0.001			
Physician’s overall judgment				
Stable disease	(ref)	(ref)	(ref)	
Slow progression	−5.46 −12.48; 1.55 0.126	−0.42 −1.61; 0.76 0.484	2.59 −3.36; 8.54 0.392	
Rapid progression	−15.28 −25.42; −5.14 0.003	−2.74 −4.73; −0.75 0.007	9.06 1.35; 16.76 0.021	
No judgement possible	−5.43 −12.72; 1.86 0.144	−0.43 −1.68; 0.81 0.495	2.40 −3.98; 8.77 0.460	
Long-term oxygen therapy	−14.31 −20.66; −7.95 <0.001	−3.20 −4.32; −2.08 <0.001	7.42 1.74; 13.10 0.011	22.99 13.50; 32.48 <0.001
NYHA functional class				
I	(ref)		(ref)	(ref)
II	−8.52 −15.67; −1.37 0.020		12.85 5.76; 19.94 <0.001	15.78 7.55; 24.01 <0.001
III	−7.40 −15.36; 0.55 0.068		21.05 13.06; 29.04 <0.001	28.96 18.96; 38.95 <0.001
IV	−24.87 −37.41; −12.32 <0.001		29.63 19.32; 39.94 <0.001	38.49 22.48; 54.51 <0.001
FVC %pred		0.04 0.01; 0.07 0.006	−0.21 −0.36; −0.06 0.005	−0.33 −0.57; −0.09 <0.001

Considered variables in stepwise multivariable linear regression analyses: Disease duration, FVC %pred, Long-term oxygen therapy, Age, Physician’s overall judgment, NYHA stage, Duration since first symptoms, GAP index, No. of comorbidities, 6MWD, Sex, Hospitalisation in last 12 months, Pulmonary rehabilitation, CPI

Both the EQ-5D index and the EQ-5D TTO were statistically significantly associated with LOT, the 6-MWD and the NYHA functional class (II, III, and IV). The WHO-5 was associated with LOT, and NYHA class III and IV. Finally, the UCSD SoB was associated with LOT, and NYHA class and %FVC.

## Discussion

Idiopathic pulmonary fibrosis (IPF) is not only a severe life-shortening disease; it also significantly impairs patients’ quality of life. In this study, we present data from a large cohort of IPF patients. To our knowledge, this is one of the first-presentations of such data collected under real-world conditions. Overall, impairment in QoL and symptom burden were immense.

Compared to a very recent report from the Australian IPF registry, QoL impairment was very similar with a SGRQ total score of 46.6 (and 48.3 in our registry). Similarly, to the data presented here, an association between QoL and dyspnoea and physiological data were reported. Yet, in contrast to our analyses also cough and depression were major contributors to diminished QoL – reasons for this may be explained by different tools used to assess depression (HADS) and a structured tool to assess cough severity [18]. Another, yet retrospective very recently published cohort of 182 IPF patients reported an association between the SGRQ total score and overall survival [19]. In comparison to recently-completed, randomised controlled drug trials, the patients in our registry had more severe QoL impairment and a higher symptom burden. For example, in the two INPULSIS trials of nintedanib, which together included 1066 patients, the mean total SGRQ score was 39.4–39.8 points in the various arms [20]; it was 48.3 in our registry. The same is true of other drug trials for IPF: ambrisentan (492 subjects: mean SGRQ total 40.5–44.5) [21], interferon gamma-1b (826 subjects: 41.6–42.4) [22]. Similarly, mean UCSD dyspnea score was higher in this registry (47.8) than participants in recently conducted trials (e.g., the ASCEND trial on pirfenidone, 555 patients, mean UCSD 34.0–36.6 points). [23] Such differences in symptoms and QoL are likely explained by differences in disease severity and baseline characteristics. For example, in our real world cohort, the burden of comorbidities known to portend a worse prognosis in IPF [3] was not insignificant, with 322 (51.7%) of our registry enrollees having at least two comorbid conditions [1]. These patients would have been excluded from most drug trials.

To our knowledge, this is the first time, investigators have assessed the association between the presence of specific comorbid conditions and QoL. We observed that comorbid conditions contribute greatly to QoL impairment. However, additional research is needed to determine if therapeutic targeting comorbidities will improve QoL in these patients. Although other investigators have assessed QoL in IPF patients under real-world conditions, they found no correlation between various baseline characteristics and QoL [24]. This may stem from a lack of power. In our cohort, SGRQ total score was higher in women than men, an observation noted by other investigators [25]. The reason for this difference is unknown but merits further investigation.

Like other investigators, we found that QoL was more impaired in patients on LTOT than in those not on LTOT. In fact, LTOT was an independent predictor of QoL even with adjustment for disease severity [24, 26]. This likely stems from the real and perceived constraints LTOT places on patients [27]. QoL impairment was also greater among patients who were prescribed anti-inflammatory therapy, anti-reflux therapy and other non-pharmacological interventions. In this observational study, causation cannot be discerned, and more research is needed to improve understanding of these results.

In several studies, investigators reported correlation coefficients between the SGRQ and one or more other patient-related assessment of health related quality of life, health status or symptoms including the Borg Dyspnea Index [26, 28], Cough Quality of Life Questionnaire [29], the Baseline Dyspnea Index [28, 30, 31], King’s Brief Interstitial Lung Disease questionnaire, [32, 33] Dyspnea Score [34], Short-Form 36 Physical Component Summary [28], Dyspnea-12 [35], and UCSD SoB questionnaire [7] among others. Overall, there were moderate to strong correlation between the SGRQ total score and the total score of these instruments [15], thus supporting the validity of the SGRQ total to capture QoL in patients with IPF. It is reassuring that data from our study mirror results from these other studies. Like them, our results support the validity of the SGRQ (and the other instruments used in INSIGHTS) for use in IPF, including a large, real-world, German cohort. In future research, shorter questionnaires with longitudinal and cross-cultural validity should be developed for use in daily patient care [32, 33].

In IPF patients, a major challenge is how to improve QoL impairment. Currently, only sildenafil, pulmonary rehabilitation or specialized, multi-modality treatment programs may have a role [36–38]. Unfortunately, the two globally-approved anti-fibrotic drugs, nintedanib and pirfenidone, have not been shown to do so. Hopefully, ongoing development and research efforts will lead to therapeutic interventions that allow IPF patients to live better with the disease.

There are limitations to our study. The QoL assessment tools we used were not originally developed for IPF, but they do have data to support their validity in this disease. Instruments such as the K-BILD [32] or A Tool to Assess Quality of Life in Idiopathic Pulmonary Fibrosis (ATAQ-IPF-cA) [39] which were developed for patients with interstitial lung disease may have reflected impairments more precisely in our cohort. However, these instruments’ psychometric properties have yet to be examined in German patients. Because all registry patients were being treated in specialized ILD centers, these results may not generalize to the larger IPF population. IPF was diagnosed at the participating centers according to current guidelines without undergoing another central MDT review which may explain some differences between the results reported here and clinical trial cohorts, although recent data suggest that experienced physicians are very accurate in diagnosing IPF [40]. Further, QoL data may have been biased in the cohort reported here as incident IPF patients were slightly underrepresented compared to patients without available HrQoL data. However, a strength of the INSIGHTS-IPF registry is that enrollees were prospectively and consecutively recruited, and it employs source data verification, statistical plausibility checks and queries.

## Conclusions

Health related quality of life is substantially impaired in patients with IPF, and drivers of this impairment include symptoms, comorbidities, LOT and disease severity. While current treatments improve the course of the disease and perhaps survival, additional investigation is needed to identify interventions that durably to improve this important outcome in IPF patients.

## Additional file

Additional file 1: Table S1. Comparison of baseline characteristics of patients with and without available QoL data (total enrolled patients *n* = 737). (DOCX 15 kb)
