# Supplementary material for: Health related quality of life in patients with idiopathic pulmonary fibrosis in clinical practice: insights-IPF registry
Source: Respir Res. 2017 Jul 14;18:139. doi: 10.1186/s12931-017-0621-y (PMC5512739; doi:10.1186/s12931-017-0621-y)
Supplement: Additional file 1: Table S1. — Comparison of baseline characteristics of patients with and without available QoL data (total enrolled patients n = 737). (DOCX 15 kb) [file 12931_2017_621_MOESM1_ESM.docx]

**Table S1. Comparison of baseline characteristics of patients with and without available QoL data (total enrolled patients n=737)**

|  |  |  |  |  |
| --- | --- | --- | --- | --- |
|  |  | Patients with HrQoL data | Patients without HrQoL data |  |
|  |  | n=623 | n=114 | P value |
|  |  |  |  |  |
|  |  |  |  |  |
| Female |  | 481 (77.2%) | 99 (86.8%) | 0.021 |
| Age, years |  | 69.6 (8.7) | 69.4 (10.5) | 0.858 |
|  |  |  |  |  |
| Body mass index, kg/m^2^ | | 27.5 (4.1) | 27.9 (4.4) | 0.323 |
|  | underweight | 4 (0.6%) | 1 (0.9%) | 0.486 |
|  | normal weight | 167 (26.8%) | 24 (21.1%) |  |
|  | overweight | 305 (49.0%) | 56 (49.1%) |  |
|  | obesity | 147 (23.6%) | 33 (29.0%) |  |
|  |  |  |  |  |
| Age at first symptom onset, years | | 65.8 (10.1) | 65.5 (12.2) | 0.770 |
| Age at IPF diagnosis, years | | 67.6 (9.6) | 67.7 (11.6) | 0.966 |
|  |  |  |  |  |
| Duration since first symptoms, years | | 3.6 (4.0) | 3.7 (4.6) | 0.860 |
| Disease duration, years | | 2.0 (3.3) | 1.8 (2.8) | 0.385 |
| Disease duration of less than 6 months | | 242 (38.8%) | 59 (51.8%) | 0.010 |
|  |  |  |  |  |
| Smoking status | |  |  | 0.411 |
|  | never | 237 (38.0%) | 37 (32.5%) |  |
|  | former stopped | 376 (60.4%) | 76 (66.7%) |  |
|  | current | 10 (1.6%) | 1 (0.9%) |  |
|  |  |  |  |  |
| Gastro-oesophageal reflux | | 192 (30.8%) | 28 (24.6%) | 0.353 |
| Genetic predisposition | | 31 (5.0%) | 6 (5.3%) | 0.804 |
|  |  |  |  |  |
| Six-minute walk distance, meters | | 272.4 (196.1) | 271.7 (191.4) | 0.972 |
|  |  |  |  |  |
| % FEV_1_ |  | 75.3 (19.4) | 75.5 (21.2) | 0.922 |
| % FVC |  | 67.5 (17.8) | 67.2 (19.3) | 0.897 |
| % DL_CO_ |  | 35.6 (17.0) | 35.4 (15.6) | 0.883 |
|  |  |  |  |  |
| GAP index |  |  |  | 0.425 |
|  | Stage I | 87 (20.2%) | 14 (14.4%) |  |
|  | Stage II | 238 (55.2%) | 58 (59.8%) |  |
|  | Stage III | 106 (24.6%) | 25 (25.8%) |  |
|  |  |  |  |  |
|  |  |  |  |  |
